# Risk factors for disruptions in tuberculosis care in Uganda during the COVID-19 pandemic

**DOI:** 10.1371/journal.pgph.0001573

**Published:** 2023-06-02

**Authors:** Peter D. Jackson, Stella Zawedde Muyanja, Isaac Sekitoleko, Mudarshiru Bbuye, Madeline Helwig, Roma Padalkar, Mariam Hammad, Dennis Hopkinson, Trishul Siddharthan

**Affiliations:** 1 Division of Pulmonary and Critical Care, Department of Medicine, Virginia Commonwealth University, Richmond, Virginia, United States of America; 2 Infectious Disease Institute, College of Health Sciences, Makerere University, Kampala, Uganda; 3 Medical Research Council/Uganda Virus Research Institute and London School of Hygiene & Tropical Medicine Uganda Research Unit, Entebbe, Uganda; 4 London School of Hygiene and Tropical Medicine, London, England; 5 Makerere University Lung Institute, College of Health Sciences, Makerere University, Kampala, Uganda; 6 Virginia Commonwealth University School of Medicine, Richmond, Virginia, United States of America; 7 Rowan University School of Osteopathic Medicine, Glassboro, New Jersey, United States of America; 8 Division of Pulmonary and Critical Care, Department of Medicine, Duke University, Durham, North Carolina; 9 Division of Pulmonary and Critical Care, Department of Medicine, University of Miami, Miami, Florida, United States of America; Universidad Peruana Cayetano Heredia, PERU

## Abstract

**Background**: During the COVID-19 pandemic, TB mortality increased while diagnoses decreased, likely due to care disruption. In March, 2020, Uganda—a country with high TB burden, implemented a COVID-19 lockdown with associated decrease in TB diagnoses. This study aims to examine patient level risk factors for disruption in TB care during the COVID-19 pandemic in Uganda. This retrospective cross-sectional cohort study included six TB clinics in Uganda. Clustered sampling included phases of TB care and three time-periods: pre-lockdown, lockdown and post-lockdown. Characteristics of patients with TB care disruption (TBCD), defined as those with > 2 months of symptoms prior to diagnosis or who missed a TB clinic, and those without TB care disruption (non-TBCD) were analyzed between time-periods. 1,624 charts were reviewed; 1322 were contacted, 672 consented and completed phone interview; pre-lockdown (n = 213), lockdown (n = 189) and post-lockdown (n = 270). TBCD occurred in 57% (385/672) of patients. There was an increase in the proportion of urban patients in the TBCD and non-TBCD groups during post-lockdown (p <0.001). There was no difference in demographics, HIV co-infection, socioeconomic status, or distance to TB clinic between TBCD and non-TBCD groups or within TBCD by time-period. There were few differences amongst TBCD and all TB patients by time-period. The increase in urban patients’ post-lockdown may represent a portion of urban patients who delayed care until post-lockdown. Insignificant trends suggesting more TBCD amongst those who lived further from clinics and those without HIV-coinfection require more investigation.

## Introduction

The COVID-19 pandemic severely disrupted healthcare globally. This is particularly concerning in low-middle income countries (LMICs), where health resources are limited and where vaccination has lagged [1]. The annual incidence of tuberculosis (TB) is 10 million annually and 1.5 million deaths per year are related to TB; most in LMICs. Prior to COVID-19, TB was the leading cause of infectious death in the world [2]. In 2020 deaths from TB increased for the first time in 18 years, while the number of overall TB diagnoses decreased with an estimated 1.4 million excess deaths and 6.3 million excess cases over 5 years [3–5]. The excess mortality is thought to stem primarily from disruptions in TB care and delays in diagnosis during mitigation measures, such as lockdowns and transportation closures [6].

Disruptions in TB care are significant for several reasons. First, delays in diagnosis and care disruption are associated with a notable increase in all-cause mortality for TB patients [7–9]. Individuals without disruptions in care have a higher quality of life, when measured with validated tools, and less risk of developing multi-drug resistant tuberculosis [10]. Finally, delays in diagnosis of TB and interruptions in treatment likely effect transmission of TB; studies suggest that robust TB programs with timely diagnosis and high treatment adherence lead to lower rates of new TB infection [3, 10, 11].

Uganda is an LMIC in East Africa with a high prevalence of TB [2]. In mid-March 2020, Uganda began a series of mitigation measures for COVID-19 with variable durations these included: suspension of public gatherings March, 18^th^, 2020—May, 18^th^, 2020, primary school closure March, 20^th^, 2020 –January, 10^th^, 2022, suspension of public transport, including motorcycle taxis and buses as well as restriction of private transport March, 25^th^, 2020 –May, 25^th^, 2020, strict nationwide curfew from 7pm-6:30am March, 30^th^, 2020 –May, 28^th^,2020 [12, 13]. A second 42-day lockdown was also instituted in April of 2021 with comparable, albeit less restrictive policies. During the initial lockdown period and initial phases of the pandemic TB testing platforms were repurposed to test for COVID-19 and TB clinic staff were affected by transportation restrictions and were reassigned to COVID-19 units leading to reports of understaffing [14, 15]. Clinics also employed strategies to ensure continuation of essential TB services which included: financial incentives for healthcare workers, PPE distribution and dispensing medications for multiple months during refills [16].

Similar measures to control the spread of COVID-19 were common in other LMICs and likely led to disruptions in TB care. In a study of 84 countries with high rates of TB, TB case notifications decreased by 21% after March 2020 with an even larger impact in the ten highest burden countries [17]. While these studies describe the scope of the problem there is limited primary data examining individual risk factors for TB care disruption during the COVID-19 pandemic. Studies to understand who is most at risk for TB care disruption may inform public health interventions aimed at maintaining TB care continuity while the world continues to combat the COVID-19 pandemic.

## Methods

**Fig 1:**
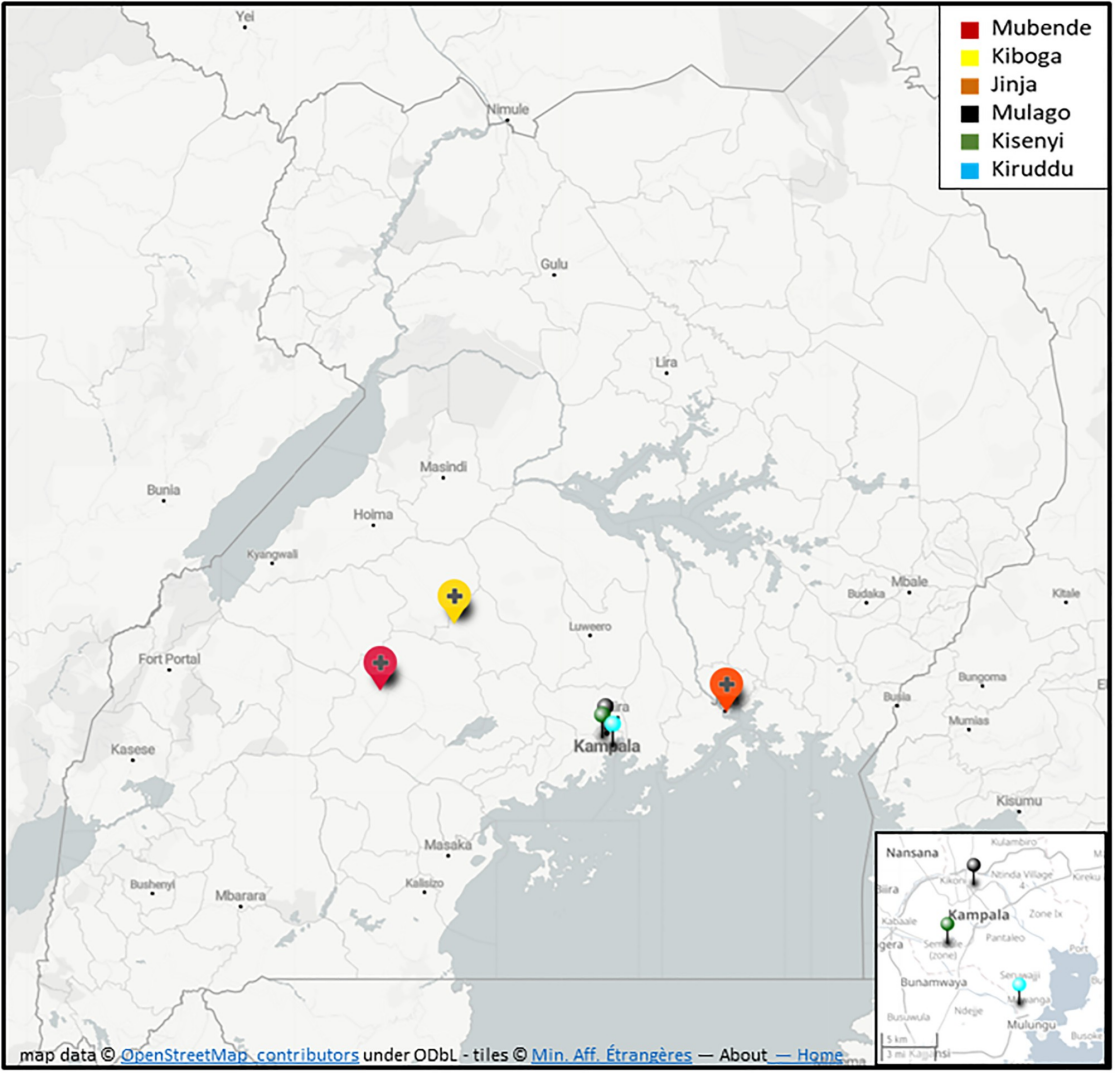
Map of TB clinic locations. Figure created using Umap https://umap.openstreetmap.fr/en/ and open source platform utilizing OpenStreetMap licensed under the Open Data Commons Open Database License (ODbL) by the OpenStreetMap Foundation (OSMF).

To assess factors associated with TB care disruption (TBCD) in Uganda during transportation lockdowns we classified patients into two groups: those that reported a care delay (> 2 months from symptoms onset to diagnosis or admission to TB-wards) or those that missed at least one clinic visit during treatment (> 3 days from scheduled visit) were assigned to the TBCD group. Those who had diagnosis < 2 months prior to symptoms and no missed visits were considered non-TBCD patients. Participants were over the age of 18 and had a diagnosis of TB from chart review at six TB clinics (three urban and three rural) who received care between September 2019–2020 in Uganda. Rural clinics included: Jinja Regional Referral Hospital, Mubende Regional Referral hospital and Kiboga General Hospital. Urban clinics included: Kiruddu National Referral Hospital-Kampala district, Kisenyi Health Centre IV-Kampala district and Mulago National Referral Hospital-Kampala district. (Fig 1) In attempt to obtain a representative sample these clinics represented urban and rural care centers as well as a range of smaller referral hospitals (Kiboga, Kisenyi) and the largest national referral hospitals (Mulago, Kirrudu). Our sampling plan involved a cross-sectional, clustered sampling methodology to ensure adequate recruitment of subjects who had experienced different TB care events during each time-period: pre-lockdown (September, 1st, 2019 –March, 15^th^, 2020), lockdown (March, 16^th^, 2020—June, 1^st^, 2020) and post-lockdown (June, 2^nd^, 2020 –September, 1^st^, 2020) was utilized. Dates for lockdown were chosen to focus on transportation lockdown periods with the end date of the 1^st^ of June to coincide with the first Monday following easing of public transport restrictions. Data on date of diagnosis, admission to TB ward, clinic attendance and missing clinic was collected. Only charts with complete data were included in the analysis.

Sociodemographic data, time from symptom onset to TB care, comorbid conditions, smoking and exposure history, medication and clinic adherence and perceived barriers to care were collected by phone. Quality of life was also measured via phone interview using the EQ-5D-3L, a well validated tool which has been used frequently in TB research and measures quality of life in five dimensions; the St. George Respiratory Questionnaire (SGRQ), a respiratory quality of life questionnaire consisting of 76 items in three categories has been validated for use in Uganda and is also validated in TB patients [18–21]. To mitigate selection bias, individuals who did not answer were called three times on different days, and family contact numbers within the charts were also called to attempt to reach them.

To determine factors associated with TBCD, we compared those who reported > 2 months of symptoms prior to TB diagnosis or a missed clinic visit during phone interview with those with no missed visit or care delays (non-TBCD). Potential risk factors for TBCD were compared amongst groups and time-periods and included: Demographics, distance from residence to clinic (measured as distance from patient’s parish to TB clinic), co-morbidities (including co-infection with HIV), socioeconomic status (SES), employment status, urban or rural TB clinic and time of TB care (pre-lockdown, lockdown or post-lockdown see above).

Socioeconomic status was determined utilizing a modified WAMI score. The WAMI score was computed based on ownership of household assets, water source/sanitation facilities, and household income [22]. This SES score variable has been described previously and validated in LMIC settings including Uganda. Principal component analysis was conducted on a correlation matrix of selected variables and the first principal component was used as the basis for a SES score for each individual, these scores were then divided into three SES categories (low, middle and high income).

### Statistical analysis

Categorical variables were summarized using frequencies and percentages. Continuous variables were summarized using median and interquartile ranges (IQR). The chi-square and Fisher’s exact test were used to assess the association between the categorical variables and the outcomes (i.e care disruption as well as comparisons across the three time-periods). Kruskal Wallis test was used to compare medians for continuous variables by the outcome variable. Additionally, using a causal approach, univariable and multivariable logistic regression models were fitted to estimate the effect of each explanatory variable and the outcome. From the fitted models, odds ratios and 95% confidence intervals were then reported. All tests were done at 5% level of significance and analysis was done using Stata version 17.

We calculated that 346 participants would be needed in our reference group (pre-lockdown) and 173 in our lockdown and post lockdown groups to obtain 80% power to detect a difference of 15% with .05 alpha.

Regulatory approval was obtained from the Makerere University School of Medicine Research and Ethics Committee of the College of Health Sciences (Ref: MakSOMREC 2021–54 and the Virginia Commonwealth University IRB (HM20020265). All subjects who completed phone interviews provided verbal witnessed consent via phone in accordance with local regulations regarding human subject interviews.

## Results

### Parent sample

**Fig 2:**
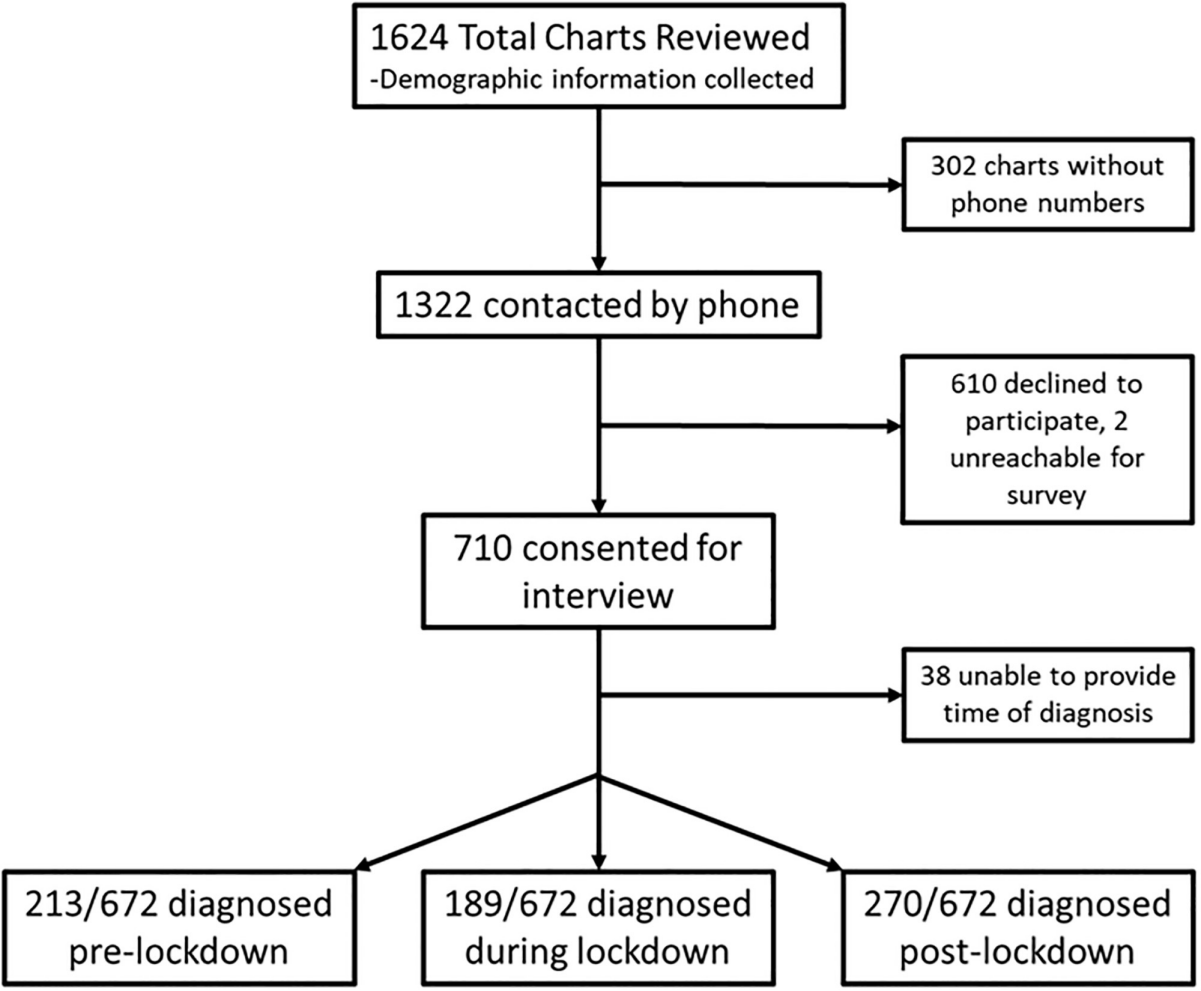
Consort diagram summarizing data collection and exclusion.

1,624 charts were reviewed from patients at six TB clinics throughout Uganda who obtained care from September 2019 to September 2021; the median age for all charts reviewed was 34 (IQR 26–44) and a majority were male 63% (1023/1624); Phone numbers were available for 1322 individuals and 1320 were contacted, 2 individuals phone numbers were not in service and family member numbers were not answered after 3 attempts. A total of 54% (710/1320) of TB patients consented and underwent phone interview and 672 completed interviews (Fig 2) which did not meet our predefined enrollment. The demographics from the parent sample and amongst those undergoing phone interviews were not different in regards to age, HIV status, urban-rural location, or gender (p > 0.05 for each comparison).

### Comparison of TBCD and non-TBCD groups from all time-periods

There was a high rate of TBCD in our phone interview sample 57.2% (385/672). There were no differences in gender, age or comorbid conditions, including HIV co-infection amongst the aggregated TBCD group when compared to the non-disruption group (p >.05 for all comparisons). There was no difference in the distance of patient residence from TB clinic in those with TBCD vs. those without TBCD (4.5km (IQR 2.3-9km) vs. 6 (IQR 3-11km, p = 0.097). There was a weak evidence suggesting fewer subjects with HIV-coinfection experiencing TBCD compared to those without HIV-coinfection overall, 51.6% vs. 58.6% (p = 0.12), however this was not significant. Socioeconomic status also did not differ significantly among all patients with TBCD and those without TBCD. Results were similar in the logistic regression model.

### Comparison of groups by time-period

**Fig 3:**
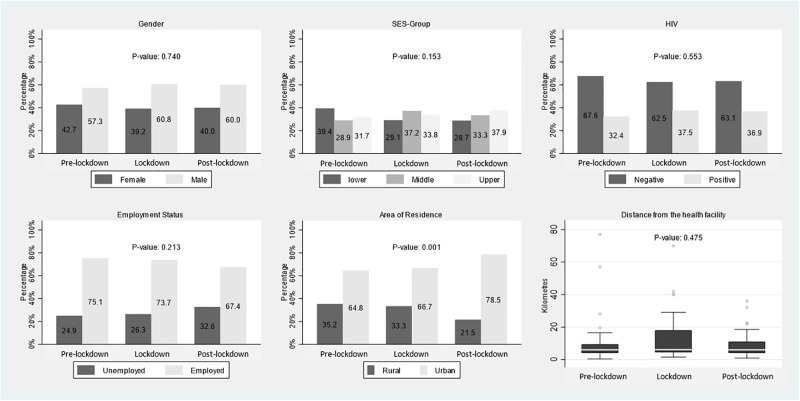
Histogram of characteristics of all TB patients by time-period.

**Fig 4:**
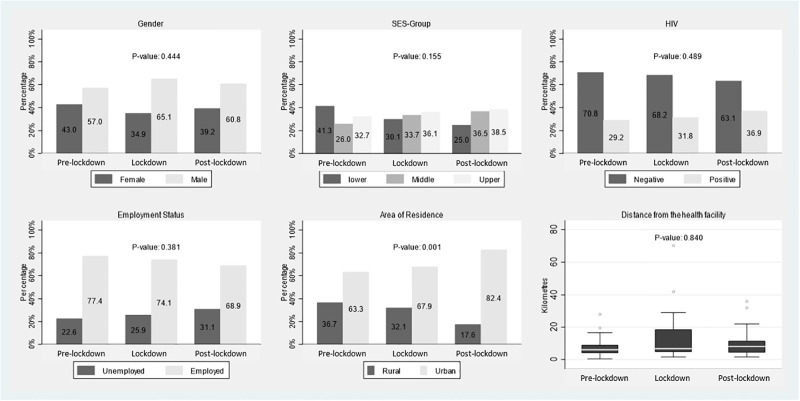
Histogram of characteristics of patients with TB care disruption by time-period.

There was no difference in the number of people who experienced TBCD by time-period (pre-lockdown 128/213 (60.1%), lockdown 109/189 (57.7%) and post-lockdown 98/170 (57.6%) (p = 0.84) (Table 1). We also analyzed the proportion of people experiencing only care delay over 2 months from symptom onset to diagnosis or admission by time-period and found no difference (pre-lockdown 62/80 (77.5%), lockdown 80/106 (75.5%), post-lockdown 82/108 (76.0%) (p = 0.946). When comparing the difference in distance from TB clinic amongst the TBCD group by time-period there was no significant difference (p = 0.86) (Tables 1 & 2). Rates of HIV-coinfection amongst those with TBCD was not significantly increased from pre-Lockdown 29.3% vs lockdown 31.8% and post-lockdown 36.9% (p = 0.49) (Table 2). The SES of all TB-patients by time-period showed a non-significant reduction in the number of lower SES patients who engaged in care during lockdown (29.1%) and post-lockdown (28.7%) compared with (39.4%), (p = 0.153) (Table 1, Fig 3) Socioeconomic status also did not differ by time-period in our TBCD group (p = 0.155), (Table 2, Fig 4).

**Table 1:** Characteristics of all participants by time-period.

Chart Review: 1624 Charts Total					
Age in years, median (IQR)	34 (26–43)				
Gender:	Female: 37% (601/1624), Male 63% (1024/1623)				
Interviewed Sample: 672 Completed Interviews					
Variables	Overall (n = 672)	Pre-lockdown (n = 213)	Lockdown (n = 189)	Post-lockdown (n-270)	P-values
**Age in years, median (IQR)**	33 (26–42)	32 (25–42)	33 (27–40)	34.5 (25–42)	0.090*
**Gender**					
Female	273(40.6)	91(42.7)	74(39.1)	108(40.0)	0.740**
Male	399(59.4)	122(58.3)	115(60.9)	162(60.0)	
**Distance to TB care (Km), median (IQR)**	5.6 (3.0–11)	6 (3–11)	5 (2.7–10)	5.2 (3.0–11)	0.475*
**Area of residence**					
Rural	196(29.2)	75(35.2)	63(33.3)	58(21.5)	0.001**
Urban	476(70.8)	138(64.8)	126(66.7)	212(78.5)	
**HIV status (148 responses missing)**					
Negative	338(64.5)	125(67.6)	95(62.5)	118(63.1)	0.553**
Positive	186(35.5)	60(32.4)	57(37.5)	69(36.9)	
**Employment Status (148 responses missing)**					
Unemployed	147(28.1)	46(42.9)	40(26.3)	61(32.6)	0.213**
Employed	377(71.9)	139(75.1)	112(73.7)	126(67.4)	
**Quantiles of SES (170 responses missing)**					
Lower	164(32.7)	71(39.4)	43(29.1)	50(28.7)	0.153**
Middle	165(32.9)	52(28.9)	55(37.2)	58(33.3)	
Upper	173(34.4)	57(31.7)	50(33.7)	66(38.0)	

TB: Tuberculosis, Km: Kilometers, IQR: Interquartile Range, HIV: Human Immunodeficiency Virus, SES: Socioeconomic status

*Kruskal Wallis Test used,

**Chi-Squared Test used

**Table 2:** Characteristics of TB patients with TBCD.

Variables	Overall: 385/672 (57.3%)	Pre-lockdown: 128/213 (60.1%)	Lockdown: 109/189 (57.7%)	Post-lockdown 148/270 (54.8%)	P-values
**Age in years, median (IQR)**	34 (26–43)	33 (26–45)	32 (27–41)	35 (25–43)	0.147*
**Gender**					
Female	151 (39.2)	55 (43.0)	38 (34.9)	58 (39.2)	0.444**
Male	234 (60.7)	73 (57.0)	71 (65.1)	71 (65.1)	
**Distance to TB care (KM), median (IQR)**	6 (3.4–11)	6 (4–11)	5 (3.3–10)	6 (3.4–11)	0.84*
**Area of residence**					
Rural	108 (28.1)	47 (36.7)	35 (32.1)	26 (17.6)	0.001**
Urban	277 (71.9)	81 (63.3)	74 (67.9)	122 (82.4)	
**HIV status (91 responses missing)**					
Negative	198 (67.4)	75 (70.8)	58 (68.2)	65 (63.1)	0.489**
Positive	96 (32.7)	31 (29.3)	27 (31.8)	38 (36.9)	
**Employment Status (90 responses missing)**					
Unemployed	79 (26.5)	24 (22.6)	22 (25.9)	32 (31.1)	0.381**
Employed	216 (73.5)	82 (77.4)	63 (74.1)	71 (68.9)	
Quantiles of SES* (102 responses missing)					
Lower	92 (32.5)	43 (41.4)	25 (30.1)	24 (25.0)	0.155**
Middle	90 (31.8)	27 (26.0)	28 (33.7)	35 (36.5)	
Upper	101 (35.7)	34 (32.7)	30 (36.1)	37 (38.5)	

TB: Tuberculosis, Km: Kilometers, IQR: Interquartile Range, HIV: Human Immunodeficiency Virus, SES: Socioeconomic status

*Kruskal Wallis Test used,

**Chi-Squared Test used

### Comparison of rural and urban TB patients by time-period

Our sample had more patients from urban settings than rural settings for all time-periods (Figs 3 and 4). There was an increase in the proportion of TB patients overall and those with TBCD from urban areas in the post-lockdown period (p <0.001) for both comparisons (Table 2). This proportion was similar in TBCD and non-TBCD patients.

## Discussion

In this retrospective clustered cross-sectional study, we were able to collect a large sample of patients who had received care for TB before, during and after the initial COVID-19 lockdown in Uganda. While previous reports have utilized TB registrar data to describe the reduction in TB case notifications, this study is one of the first to use interviews to identify patient level risk factors for TBCD during the pandemic [17, 23]. Despite our large sample and high telephone contact rate (approximately 60%) we did not demonstrate many significant differences amongst those with TBCD before the COVID-19 lockdown, during the lockdown or after the initial lockdown period.

Research prior to 2020 has shown clinical and system-related factors associated with TB diagnostic delay [24–28]. The most consistent associations with a delay in TB diagnosis have included: low socioeconomic status, rural location or longer distance from TB clinics, poor knowledge about TB, substance abuse, advanced age and seeking care from traditional healers [24]. Previous work in Uganda and East Africa demonstrated that the most common risk factors for diagnostic delay were male gender, presentation to a public clinic and poor TB knowledge [29, 30]. Data on factors associated with interruptions in TB care after diagnosis in Africa have shown numerous characteristics associated with disruption in care such as: HIV co-infection (both protective and predictive of disruption), distance from a TB center, previous TB diagnosis, TB stigma and cost of TB treatment [25, 26, 31].

While the present study did not identify a significant increase in those reporting delays in diagnosis in the lockdown period, there were higher numbers of urban patients seeking care post-lockdown. The COVID-19 lockdowns were likely to be more strictly enforced in urban areas where police presence is greater; this may have led to a “backlog” of urban patients who sought care after the initial COVID-19 lockdown was lifted. The lack of other differences between time-periods for those with TBCD was surprising and but was not entirely inconsistent with other work in countries outside of Africa that examined TB care during the COVID-19 pandemic. A retrospective Chinese study examining medical records of TB patients from a national database from TB patients during the 10 weeks prior to COVID-19 lockdown, 10 weeks during COVID-19 lockdown and 10 weeks following lifting of lockdown found that TB notifications dropped by 60% during lockdown and individuals presented with more severe disease on chest x-ray during lockdown compared to pre and post-lockdown. However, there was no difference in time to diagnosis when comparing the 10-week periods pre, during or post-lockdown although there was a longer delay in care for all time-periods in 2020 when compared to historical control periods [32].

We hypothesized that patients with TBCD would live further from TB clinics than those who did not have TBCD. We also believed that all subjects, regardless of TBCD, who did engage in care during lockdown would have a shorter distance to care than those who received care prior to lockdown. While our analysis demonstrated weak evidence that TB patients after the COVID-19 lockdown lived a greater distance from TB care, this was not statistically significant; furthermore, there was no change in distance by time-period for the TBCD group. Our data showed weak evidence towards less care disruption overall in TB-HIV coinfection but this was not significant and not affected by time-period. Further investigation into these groups may be reasonable to evaluate if better engagement in care and robust systems to deliver care to HIV patients during the COVID-19 pandemic improved TB care in those with coinfection.

Strengths of the study included a relatively large sample of charts at six TB clinics representing a range of settings, a high phone contact rate (approximately 80%) and obtaining interview data in 710 subjects. Uganda has a well-established TB screening and treatment infrastructure. The selected clinics represent a large study catchment area with active community surveillance and referral programs in place [33]. We additionally assessed care in urban and rural areas. There are noted differences in care delivery and access in urban vs rural settings and we did find a higher proportion of patients in the post-lockdown period from urban areas. There were a number of limitations to the present study. First, the response rate to phone calls was 53.7%. This may represent selection bias if systematic differences in the characteristics of individuals who were eligible and participated in the study differed from those who were eligible but did not participate in the study. We were unable to visit participants in person due to COVID restrictions and sampling by telephone could bias the sample towards individuals within higher income groups that do not work during the daytime. Second, interviews by phone led to missing data for some questions due to dropped calls or incomplete interviews, this provided reduced power to detect a difference in HIV status, employment status and socioeconomic status. This missing data may have produced systematic bias as individuals with lower SES, demanding employment or comorbid conditions may have been less able to complete full interviews. Lastly, splitting the sample amongst multiple time-periods reduced our power to detect significant differences between groups as well.

Ultimately this study did not identify patient level characteristics associated with TBCD during the COVID-19 lockdown in Uganda. Furthermore, there were no statistically significant differences in the demographics, SES, distance or comorbid conditions of patients who obtained care before, during or after the COVID-19 lockdown. It is important to note that there were likely disruptions in TB care, evidenced by decreased TB case notifications during the COVID-19 pandemic within Uganda and most of Africa. However, in this study none of the patient level variables investigated were associated with higher risk of TBCD or having a delay in TB diagnosis. While this is surprising it may suggest that the effect of COVID-19 mitigation measures was widespread and affected a broad range of TB patients in Uganda. It is possible that additional variables that we did not investigate may be associated with TBCD during the COVID-19 pandemic. Lastly, the fact that this study relied on patient interviews for those who had disruptions in care and may not have reliable phone access raises a concern for selection bias that excludes more subjects who experienced care disruption and we may have underestimated the overall rates of TBCD; however, policies aimed to limit infectious risk during the study period precluded other methods for patient recruitment. Further work is necessary to determine if some of the weak evidence seen in this data may represent meaningful associations with TBCD and studies with in person case finding, larger sample sizes and a focus on comparison such TBCD in those without HIV coinfection and those that live further from care may be warranted.

## Supporting information

S1 Data De-identified data set. (CSV)
